# Fibrinogen/Albumin Ratio Index Is an Independent Prognosis Predictor of Recurrence-Free Survival in Patients After Surgical Resection of Gastrointestinal Stromal Tumors

**DOI:** 10.3389/fonc.2020.01459

**Published:** 2020-08-18

**Authors:** Xianglong Cao, Jian Cui, Tao Yu, ZiJian Li, Gang Zhao

**Affiliations:** ^1^Department of Gastrointestinal Surgery, National Center of Gerontology, Beijing Hospital, Beijing, China; ^2^Institute of Geriatric Medicine, Chinese Academy of Medical Sciences, Beijing, China

**Keywords:** gastrointestinal stromal tumors, fibrinogen, albumin, fibrinogen-to-albumin ratio index, prognosis

## Abstract

**Background:** Nutritional status, systemic inflammation, and coagulation mechanism are closely related to tumor progression. Herein, we examined the role of fibrinogen-to-albumin ratio index (FARI) in the prognosis of gastrointestinal stromal tumors (GISTs) and developed a novel nomogram predicting recurrence-free survival (RFS).

**Methods:** We retrospectively analyzed data from 357 GIST patients admitted at the gastrointestinal surgery of the Beijing Hospital from January 2008 to January 2018 and underwent curative resection. FARI was calculated as fibrinogen level (g/L) /albumin level (g/L). The cutoff point of FARI was set using the point with the largest Youden index on the receiver operating characteristic curve with the 5-years recurrence-free survival as an endpoint. We used the Kaplan-Meier approach and multivariable Cox regression model to study the impact of FARI on recurrence-free survival. Finally, we developed a nomogram based on tumor size, location, mitotic index, and FARI to predict RFS. The nomogram was assessed by calculating concordance probabilities and testing calibration of predicted RFS with observed RFS. Concordance probabilities were also compared with the National Institute of Health (NIH) risk classification system.

**Results:** The ROC curve revealed that the best cutoff point of the FARI was set as 0.08. The patients were classified into the FARI-high (≥0.08) and FARI-low (<0.08) groups. FARI was significantly associated with age, size of the tumor, NIH risk category, and Mitotic Index (all *P* < 0.05). FARI was weakly associated with NLR and PLR. FARI and PNI had a weak negative association. Multivariate analysis showed that the NIH risk category and FARI were independent prognostic predictors for worse outcomes concerning RFS in GIST patients. In the high-risk subgroup, patients with low FARI also had a more prolonged RFS than patients with high FARI (*P* < 0.05). The nomogram had a concordance probability of 0.802 (SE 0.025). Nomogram predictions were well-calibrated. Concordance probabilities of the nomogram were better than NIH risk classification system [0.802 [0.025] vs. 0.737 [0.024], *p* < 0.01].

**Conclusion:** We established that preoperative FARI is a novel serum biomarker to predict the prognosis after surgical resection of GISTs. The nomogram incorporating FARI could be used to help the decision-making of clinical treatment.

## Background

Gastrointestinal stromal tumors (GISTs) constitute the most prevalent form of sarcomas in the digestive system. GISTs usually emanate from the interstitial cells of Cajal. The incidence of GISTs in China is 19–22 per million per year (1). GISTs are neoplasms with different malignant potential ranging from very low risk to high risk. Up to now, radical surgery constitutes the first option for resectable GISTs. Although surgical procedures and targeted adjuvant therapy have markedly improved over the past decade, the long-term prognosis of patients with advanced gastrointestinal stromal tumors remains poor, and the 5-years recurrence rate after an operation is as high as 50% (2).

Since the introduction of NIH risk classification in 2002, the prediction systems of GISTs have made significant progress (3). the most widely used GIST systems are Fletcher NIH classification (3), Miettinen modified NIH classification (4), AFIP classification (5), and MSKCC nomogram (6). Presently, subsequent studies have confirmed the effectiveness of the newly modified NIH risk classification, which constitutes of the location, size, mitotic index, and rupture of the primary tumor (7). Even though the risk classification of NIH is the same, the prognosis of GIST varies greatly. Therefore, it is necessary to develop useful serum biomarkers outside the NIH system to classify the development of postoperative tumor recurrence in GIST subjects. Recent studies have shown that chronic systemic inflammation and nutritional status are strictly related to the long-term prognosis of various cancers. This is achieved by changing the signal transduction pathway in the microenvironment around tumor cells (8–11). Malnutrition is prevalent in patients with malignant tumors. Research evidence shows that hypoalbuminemia decreased BMI and decreased skeletal muscle volume are all associated with poor prognosis of malignant tumors (12, 13). Besides, scoring systems that reflect inflammatory and immune levels constituting the platelet-to-lymphocyte ratio (PLR), the prognostic nutritional index (PNI), and the neutrophil-to-lymphocyte ratio (NLR) are closely linked to the clinical outcomes of malignant cancers, including GIST (14–17).

A hypercoagulable state is associated with many solid tumors and correlates with reduced survival (18). Among the coagulation factors, fibrinogen attracts much research attention because it induces inflammatory reactions and plays a vital role in tumorigenesis and metastasis. Findings of recent studies revealed that the increase of preoperative fibrinogen leads to treatment failure or poor prognosis in many kinds of tumors, such as lung cancer (19), digestive cancers (20), and solid tumors (21). A recently published study reported that elevated preoperative fibrinogen is linked to a high risk of recurrence and poor long-term prognosis in GISTs patients (22).

The results of a recent study revealed that a novel marker named fibrinogen-to-albumin ratio index (FARI) combines coagulation with nutritional status. Moreover, FARI is used to estimate the survival of patients with esophageal tumors after an operation (23). However, whether FARI is linked to the risk of reoccurrence and long-term survival in GIST patients after an operation remains unclear. Here, we investigated whether FARI predicts postoperative recurrence in patients with GIST after undergoing radical resection.

## Patients and Methods

### Patients

From January 2008 to January 2018, 357 patients who were consecutively diagnosed with primary GIST undergoing curative surgery in the gastrointestinal surgery department of a Beijing Hospital were included. The clinical features were obtained from the medical records, operative records, and pathology reports, and evaluated as prognostic factors. The current diagnosis of GIST was confirmed by histopathological and immunohistochemical criteria, including levels of receptor tyrosine kinase KIT (CD117, c-Kit) or established on GIST 1 (DOG1). The risk of recurrence of GIST was assessed using the newly adjusted National Institute of Health (NIH) risk classification system suggested by the latest Chinese consensus guidelines of the Chinese Society of Clinical Oncology (CSCO) Expert Committee (7).

The following criteria were used to enroll patients: (1) no neoadjuvant therapy based on tyrosine kinase inhibitor (TKI) or postoperative adjuvant therapy; (2) The surgical resection reaches the R0 standard (the cutting edge of the specimen is negative); (3) physiological status based on Eastern Cooperative Oncology Group (ECOG) <3 points; (4) age 18–85 years old.

The exclusion criteria were as follows: (1) partial clinical record or incomplete hematological examination data before operation; (2) accompanied by other primary malignancies; (3) suffering from infection and non-cancer inflammatory diseases; and (4) patients who underwent parenteral nutrition support before resection.

### Clinical Interventions

Curative resection (R0) was considered as a complete macroscopic and microscopic resection. Surgical procedures were based on the corresponding version of guidelines (7). Postoperative management was the same for all patients.

### Indicators and Measurements

The basic characteristics of patients, including gender, age, ECOG score, body mass index (BMI), tumor location, NIH risk category, size of the tumor, and Mitotic index, were recorded. Data were collected from blood tests just before the operation, where the data included neutrophil, lymphocyte, and platelet counts, albumin value, and fibrinogen level in the peripheral blood. Then, we calculated the definitions of NLR, PNI, PLR, and FARI as follows: NLR, neutrophil numbers /lymphocyte numbers; PNI, albumin concentration (g/L) +5 × total lymphocyte numbers (10^9^/L); PLR, platelet numbers /lymphocyte numbers; and FARI, fibrinogen concentration (g/L)/albumin concentration (g/L). We additionally assessed postoperative complications (defined as any complications that deviated from the normal postoperative process). Surgical complications were categorized based on the Clavien–Dindo classification (24). No patients died within 30 days after operation in this study.

### Follow-Up

We followed up the patients every 3–6 months up to 2 years after surgery, then every 6–12 months up to 5 years, and after that every year or until death. Physical examination, routine peripheral blood tests, imaging examinations of the abdomen including ultrasonography, computed tomography (CT) or magnetic resonance imaging (MRI), and endoscopy where needed, were performed at each visit, which was also based on the surveillance, suggested in the guideline (3).

The median follow-up was 56 months (range, 2–131), and the last follow-up date was January 2020. We designated the recurrence-free survival (RFS) as the primary endpoint, which was described the period from surgery to the time of tumor reoccurrences or metastasis. We described recurrence as evidence of disease reoccurrence, as revealed by either the computed tomography scan or the magnetic resonance image. We censored the patients who were alive without evidence of reoccurrence on the last follow-up date or who died without evidence of tumor reoccurrence.

### Statistical Analysis

According to the receiver operating characteristic (ROC) curve, we used the maximum value of the Youden index (sensitivity + specificity −1) to establish the optimal cutoff values of PNI, PLR, NLR, and FARI for predicting 5-years RFS (25). Then the areas under the curve (AUC) were calculated to compare the predicted values of these biomarkers.

We divided the patients into two classes as per the cutoff value of the FARI. We used the chi-square test in comparing the clinical characteristics between the two groups. The mean ± standard deviation is used to present all the quantitative data in this study.

We analyzed the survival curves as per the Kaplan-Meier method and compared using the log-rank test. We conducted univariate as well as multivariate analyses of survival using the Cox proportional hazards model with the stepwise forward approach for variable selection. We reported the hazard ratios (HR) computed from the Cox analysis as relative risks with correspondent 95% confidence intervals (CIs). The significant independent factors for RFS selected by multivariate COX regression analysis were used to construct a nomogram. To assess the performance of the nomogram, we used a calibration curve with the bootstrapping method to illustrate the association between the actual 5-Years RFS and the nomogram-Predicted Probability of 5-Years RFS. The predictive value of the nomogram model was evaluated with the Harrell's concordance index (C-index) and compared with the modified NIH risk classification. A two-sided *P* < 0.05 was accepted as statistically significant. The statistical analyses were computed in SPSS, version 26.0 (SPSS, IL, USA), GraphPad Prism 8.0 software (GraphPad, La Jolla, CA), and R (version 4.0.2). The R packages rms, mstate, survival, Hmisc, and ggplot2 (available at URL: http://cran.r-project.org/web/packages/) were used.

## Results

### Patient and Tumor Characteristics

The clinical characteristics are listed in Table 1. We enrolled 357 patients (185 (51.8%) males and 172 (48.2%) females) according to the inclusion criteria. The average age of the patients was 60.88 ± 12.05 (range, 24–85) years. Moreover, 15 (4.2%) cases had a BMI lower than 18.5 kg/m^2^.

**Table 1:** Relationship between clinical characteristics and Fibrinogen–Albumin Ratio Index.

**Variables**	**Total (n = 357)**	**FARI-low (n = 243)**	**FARI-high (n = 114)**	**χ^**2**^**	***P*-value**
Gender				0.795	0.373
Male (%)	185 (51.8)	122 (50.2)	63 (55.3)		
Female (%)	172 (48.2)	121 (49.8)	51 (44.7)		
ECOG score				3.621	0.164
0 (%)	267 (74.8)	189 (77.8)	78 (68.4)		
1 (%)	78 (21.8)	47 (19.3)	31 (27.2)		
2 (%)	12 (3.4)	7 (2.9)	5 (4.4)		
Age, years	60.88 ± 12.05	59.47 ± 12.56	62.82 ± 10.72	−2.466	0.014
BMI (kg/m^2^)				0.533	0.465
<18.5 (%)	15 (4.2)	12 (4.9)	3 (2.6)		
≥18.5 (%)	342 (95.8)	231 (95.1)	111 (97.4)		
Tumor size				32.394	<0.001
≤ 2 (%)	82 (22.9)	67 (27.6)	15 (13.1)		
>2, ≤ 5 (%)	142 (39.8)	105 (43.2)	37 (32.6)		
>5, ≤ 10 (%)	80 (22.4)	51 (21.0)	29 (25.4)		
>10 (%)	53 (14.9)	20 (8.2)	33 (28.9)		
Tumor location				4.126	0.248
stomach (%)	221 (61.9)	158 (65.0)	63 (55.3)		
Intestine (%)	75 (21.0)	45 (18.5)	30 (26.3)		
Colorectum (%)	27 (7.6)	19 (7.8)	8 (7.0)		
E-GIST (%)	34 (9.5)	21 (8.7)	13 (11.4)		
Mitotic index (per 50 HPF)				10.462	0.005
≤ 5 (%)	211 (59.1)	157 (64.6)	54 (47.4)		
>5, ≤ 10 (%)	60 (16.8)	38 (15.6)	22 (19.3)		
>10 (%)	86 (24.1)	48 (19.8)	38 (33.3)		
NIH risk category				22.978	<0.001
Very low (%)	64 (17.9)	54 (22.2)	10 (8.8)		
Low (%)	101 (28.3)	75 (30.9)	26 (22.8)		
Intermediate (%)	61 (17.1)	44 (18.1)	17 (14.9)		
High (%)	131 (36.7)	70 (28.8)	61 (53.5)		
Surgery				15.342	<0.001
Open (%)	184 (51.5)	108 (44.4)	76 (66.7)		
Laparoscopy (%)	173 (48.5)	135 (55.6)	38 (33.3)		
Clavien–Dindo grade				0.009	0.925
<3 (%)	314 (88.0)	214 (88.1)	100 (87.7)		
≥3 (%)	43 (12.0)	29 (11.9)	14 (12.3)		
NLR				6.111	0.013
Low (%)	96 (26.9)	75 (30.9)	21 (18.4)		
High(%)	261 (73.1)	168 (69.1)	93 (81.6)		
PLR				15.270	<0.001
Low (%)	147 (41.2)	117 (48.1)	30 (26.3)		
High (%)	210 (58.8)	126 (51.9)	84 (73.7)		
PNI				38.956	<0.001
Low (%)	147 (41.2)	73 (30.0)	74 (64.9)		
High (%)	210 (58.8)	170 (70.0)	40 (35.1)		

*FARI, fibrinogen–albumin ratio index; ECOG, Eastern Cooperative Oncology Group; BMI, body mass index; NIH, National Institute of Health; NLR, neutrophil-to-lymphocyte ratio; PLR, platelet-to-lymphocyte ratio; PNI, prognostic nutritional index*.

The most common location of the tumor was the stomach 221(61.9%), then the small intestine 75 (including duodenum, 21.0%), colon or rectum 27(7.6%), and extra-gastrointestinal stromal tumors 34(E-GIST, 9.5%), including 12 primary tumors located in the mesentery, nine in the retroperitoneum, five in the omentum, three in the liver, two in the prostate, and one in the pancreas, one in the bladder and one in the female reproductive system, respectively. No tumor rupture occurred in this study.

According to tumor size, 82 (22.9%), 142 (39.8%), 80 (22.4%), 53 (14.9%) patients were categorized into ≤ 2 cm, 2.1–5 cm, 5.1–10 cm, >10 cm groups, respectively. The median maximum tumor diameter was 4.0 cm. According to Mitotic index, 211 (59.1%), 60 (16.8%), 86 (24.1%) patients were categorized into ≤ 5, 6–10, >10 per 50 HPF groups, respectively. Based on the criteria of the modified NIH GIST risk classification, 64 (17.9%), 101 (28.3%), 61 (17.1%), and 131 (36.7%) patients were grouped into very low-, low-, intermediate-, and high-risk groups, respectively.

Based on the Clavien–Dindo classification, the rates of grade three or above complications were 12.0% (43/357).

### The Optimal Cutoff Values of FARI, NLR, PLR, and PNI for Estimating RFS

**Figure 1:**
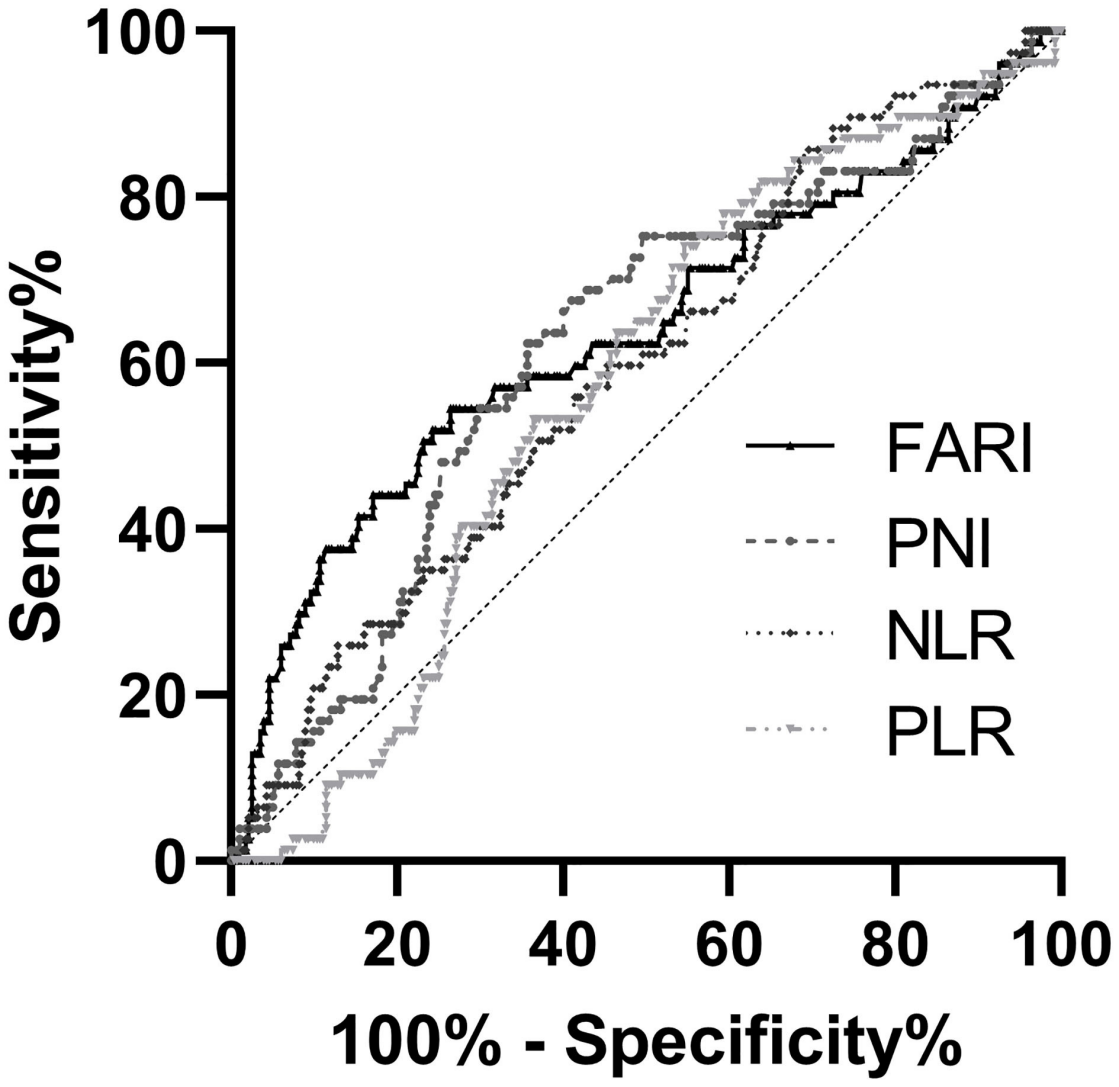
The receiver operating characteristic (ROC) analysis of NLR, PLR, PNI, and FARI. The areas under the curve (AUC) for RFS were 0.592 (*p* = 0.014), 0.572 (*p* = 0.053), 0.623 (*p* = 0.001), and 0.638 (*p* < 0.001) for NLR, PLR, PNI, and FARI, respectively. NLR, neutrophil-to-lymphocyte ratio; PLR, platelet-to-lymphocyte ratio; PNI, prognostic nutritional index; FARI, fibrinogen-to-albumin ratio index.

The median values of preoperative FARI, NLR, PLR, and PNI were 0.08, 1.44, 117.48, and 47.48, respectively. Therefore, we determined the best cutoff points of these markers for estimating a 5-years RFS via a ROC analysis approach. The areas under the curve (AUC) for RFS were 0.638 for FARI (*p* < 0.001), 0.592 for NLR (*p* = 0.014), 0.572 for PLR (*p* = 0.053), and 0.623 for PNI (*p* = 0.001), respectively (Figure 1). The best cutoff values were established 0.08 for FARI, 1.44 for NLR, 117.48 for PLR, and 47.48 for PNI, respectively.

At last, we divided the patients into two groups for subsequent analysis according to the best cutoff points as follows: a FARI-low group (<0.08, *n* = 243), or a FARI-high group (≥0.08, *n* = 114).

### The Association Between Inflammatory Markers and Clinical Characteristics

Elevated levels of FARI were markedly associated with age (χ^2^ = −2.466, *P* = 0.014), tumor size (χ^2^ = 32.394, *P* < 0.001), Mitotic index (χ^2^= 10.462, *P* = 0.005), NIH risk category (χ^2^ = 22.978, *P* < 0.001), type of surgery (χ^2^ = 15.342, *P* < 0.001), and other inflammatory markers such as NLR (χ^2^ = 6.111, *P* = 0.013), PLR (χ^2^= 15.270, *P* < 0.001), PNI (χ^2^= 38.956, *P* < 0.001), but not with gender (χ^2^ = 0.795, *P* = 0.373), BMI (χ^2^ = 0.533, *P* = 0.465), and tumor location (χ^2^ = 4.126, *P* = 0.248). The associations between the FARI and other variables are shown in Table 1.

Furthermore, FARI was weakly associated with NLR (*r* = 0.236; *P* < 0.001) and PLR (*r* = 0.259, *P* < 0.001). Besides, FARI and PNI had a significant negative correlation, but the correlation was weak (*r* = −0.325; *P* < 0.001).

### Univariate and Multivariate Survival Analyses

Out of the 357 patients, 77 patients suffered from GIST recurrence, 52 patients died of GIST recurrence, and two patients died of other diseases. Overall, the 1-year, 3-years, and 5-years RFS rates were 93.6, 84.1, and 79.1%, respectively. GIST relapsed in 77 cases (21.6%), of which 54 cases (70.1%) were high-risk patients.

**Figure 2:**
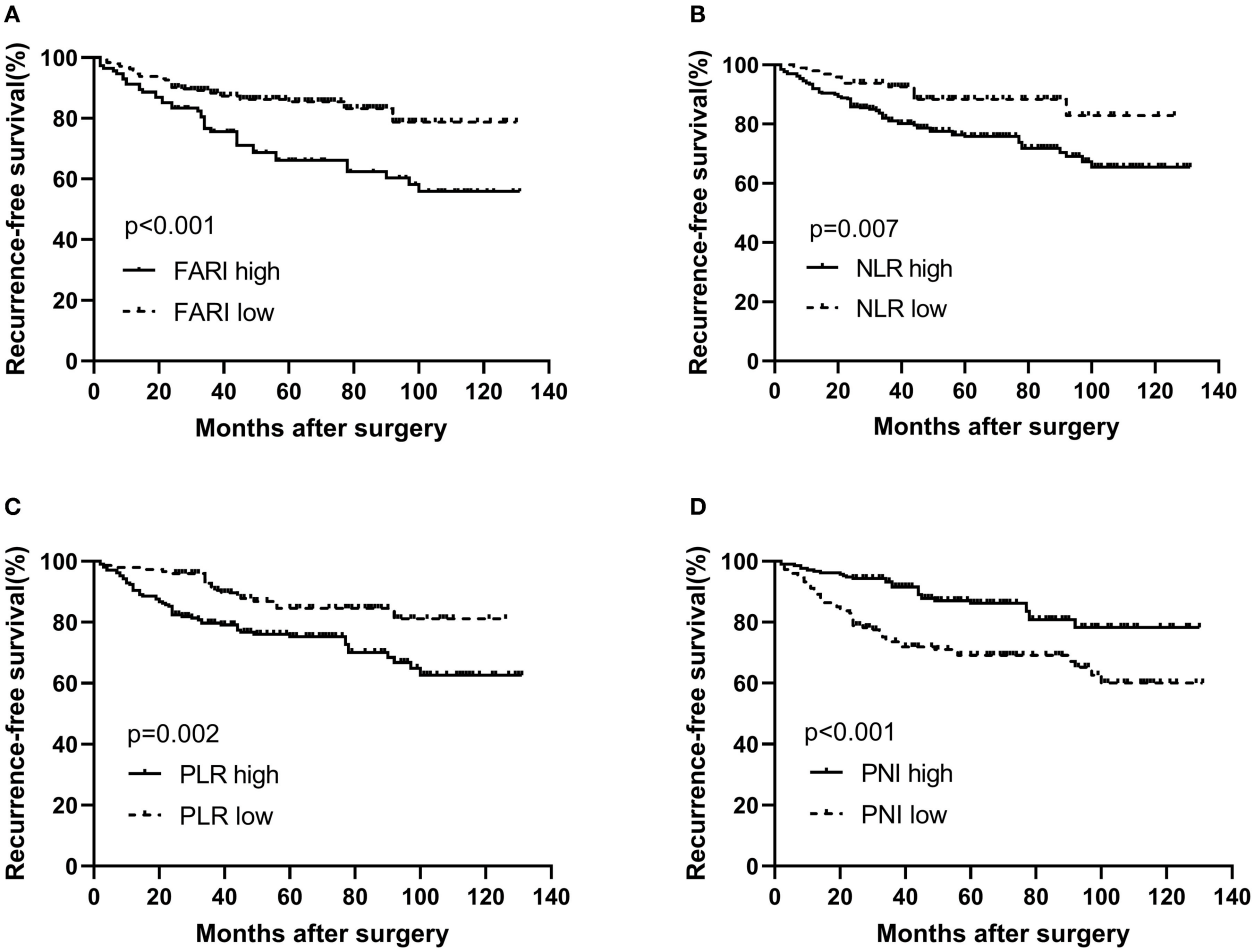
Kaplan–Meier survival curves for RFS according to FARI **(A)**, NLR **(B)**, PLR **(C)**, and PNI **(D)** in GIST patients. **(A)** The 5-years RFS rate of patients in the FARI-low group was significantly better than those in the FARI-high group (85.6 vs. 66.3%, *p* < 0.001). **(B)** The 5-years RFS rate of patients in the low-NLR group was significantly better than those in the high-NLR group (86.5 vs. 76.0%, *p* = 0.008). **(C)** The 5-years RFS rate of patients in the low-PLR group was significantly better than those in the high-PLR group (84.6 vs. 75.5%, *p* = 0.002). **(D)** The 5-years RFS rate of patients in the low-PNI group was significantly worse than those in the high-PNI group (69.3 vs. 86.3%, *p* < 0.001). NLR, neutrophil-to-lymphocyte ratio; PLR, platelet-to-lymphocyte ratio; PNI, prognostic nutritional index; FARI, fibrinogen-to-albumin ratio index.

The RFS rate in the FARI-low group was significantly higher than that in the FARI-high group (3-years RFS rate of 88.3 vs. 75.6%, 5-years RFS rate of 85.5 vs. 66.1%, *P* < 0.001, Figure 2A). The patients in the NLR-low group or PLR-low group had a remarkably more prolonged RFS compared with the patients in the NLR-high group or PLR-high group (5-years RFS rate of 85.6 vs. 75.7%, *P* = 0.007, and 5-years RFS rate of 84.4 vs. 75.2%, *P* = 0.002, Figures 2B,C). Moreover, the patients in the PNI-low group had a markedly shorter RFS compared with the patients in the PNI-high group (5-years RFS rate of 69.1 vs. 86.1%, *P* < 0.001, Figure 2D).

The findings of the univariate and multivariate investigations are listed in Table 2. In the univariate evaluations, we found tumor size (*P* < 0.001), tumor location (*P* < 0.001), mitotic index (*P* < 0.001), NIH risk category(*P* < 0.001), FARI (HR = 2.338, 95% CI: 1.492–3.663, *P* < 0.001), PNI (HR = 2.402, 95% CI: 1.518–3.801, *P* < 0.001), NLR (HR = 2.314, 95% CI: 1.222–4.381, *P* = 0.010), and PLR (HR = 2.148, 95% CI: 1.290–3.575, *P* = 0.003) to be remarkable predictors of RFS. The multivariate evaluation using the forward stepwise approach for variable selection showed that the NIH risk category (*P* < 0.001), and FARI (HR = 1.650, 95% CI: 1.047–2.601, *P* = 0.031) were independent prognostic factors of RFS.

**Table 2:** Univariate and multivariate analysis of the prognostic factors for recurrence-free survival in patients with GIST.

**Variables**	**Univariate analysis**		**Multivariate analysis**	
	**HR (95%CI)**	***P***	**HR (95%CI)**	***P***
Gender		0.350		
Male	1			
Female	0.805 (0.511–1.268)			
ECOG score		0.223		
0	1			
1	1.473 (0.880–2.466)	0.140		
2	1.774 (0.642–4.904)	0.269		
Age		0.486		
<60	1			
≥60	1.177 (0.744–1.860)			
BMI (kg/m^2^)		0.154		
<18.5	1.937 (0.781–4.808)			
≥18.5	1			
Tumor size		<0.001		
≤ 2	1			
>2, ≤ 5	1.566 (0.617–3.972)	0.345		
>5, ≤ 10	3.784 (1.534–9.337)	0.004		
>10	12.285 (5.127–29.437)	<0.001		
Tumor location		<0.001		
stomach	1			
Intestine	2.447 (1.411–4.244)	0.001		
Colorectum	3.664 (1.835–7.319)	<0.001		
E–GIST	4.037 (2.133–7.642)	<0.001		
Mitotic index		<0.001		
≤ 5	1			
>5, ≤ 10	3.962 (1.996–7.865)	<0.001		
>10	10.256 (5.769–18.232)	<0.001		
NIH risk category		<0.001		<0.001
Very low	1		1	
Low	1.683 (0.455–6.218)	0.435	1.615 (0.437–5.972)	0.472
Intermediate	3.943 (1.100–14.135)	0.035	3.591 (0.998–12.915)	0.050
High	10.681 (3.338–34.177)	<0.001	9.280 (2.877–29.937)	<0.001
Clavien–Dindo grade		0.715		
<3	1			
≥3	0.878 (0.438–1.761)			
FARI		<0.001		0.031
Low	1		1	
High	2.338 (1.492–3.663)		1.650 (1.047–2.601)	
NLR		0.010		
Low	1			
High	2.314 (1.222–4.381)			
PLR		0.003		
Low	1			
High	2.148 (1.290–3.575)			
PNI		<0.001		
Low	2.402 (1.518–3.801)			
High	1			

*FARI, fibrinogen–albumin ratio index; ECOG, Eastern Cooperative Oncology Group; BMI, body mass index; NIH, National Institute of Health; NLR, neutrophil-to-lymphocyte ratio; PLR, platelet-to-lymphocyte ratio; PNI, prognostic nutritional index; HR, hazard ratio; CI, confidence interval*.

**Figure 3:**
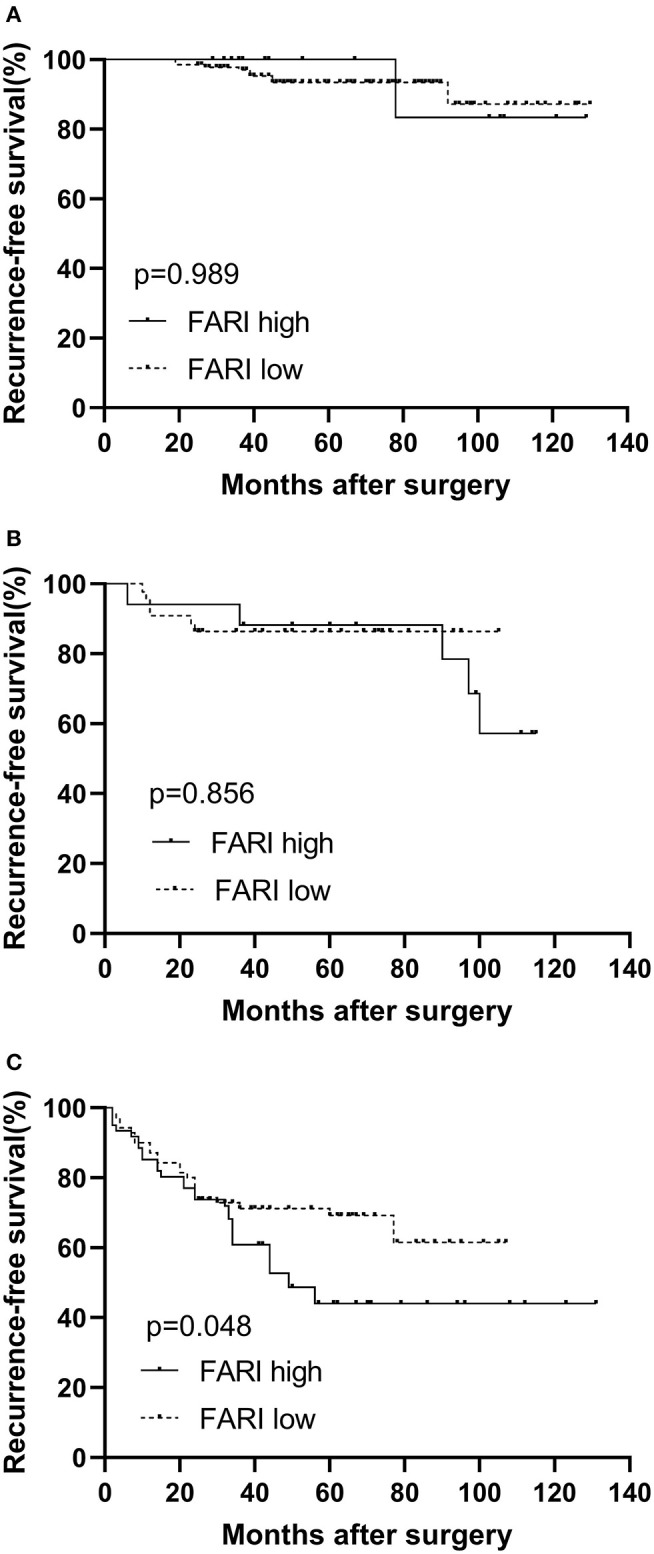
Kaplan–Meier survival curves for recurrence-free survival according to the FARI in very low & low **(A)**, intermediate **(B)**, and high risk **(C)** subgroup. A more prolonged recurrence-free survival was also observed in patients in the FARI-low group in the high-risk subgroups (*P* = 0.048), but not in the very low & low and intermediate subgroup, respectively (*P* = 0.989, 0.856). FARI, fibrinogen-to-albumin ratio index.

According to the high-risk GIST patients, the 1-year, 3-years, and 5-years RFS rates were 86.3, 66.5, and 57.3%, respectively. In the subgroup analysis, our findings indicated that more prolonged RFS was also reported in patients in the FARI-low group in the high-risk subgroups (*P* = 0.048), but not in the very low & low and intermediate subgroup (*P* = 0.989, 0.856), respectively (Figure 3).

### Construction and Validation of the Nomogram

**Figure 4:**
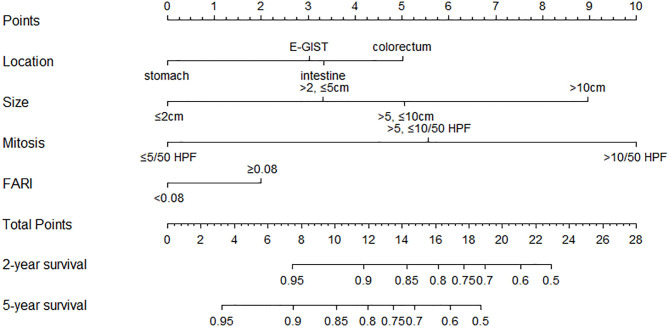
Nomogram to predict the probabilities of 2-years and 5-years recurrence-free survival of primary GIST. Points are assigned for size, mitotic index, site of origin, and FARI by drawing a line upward from the corresponding values to the “Points” line. The sum of these four points, plotted on the “Total points” line, corresponds to predictions of 2-years and 5-years recurrence-free survival (RFS).

**Figure 5:**
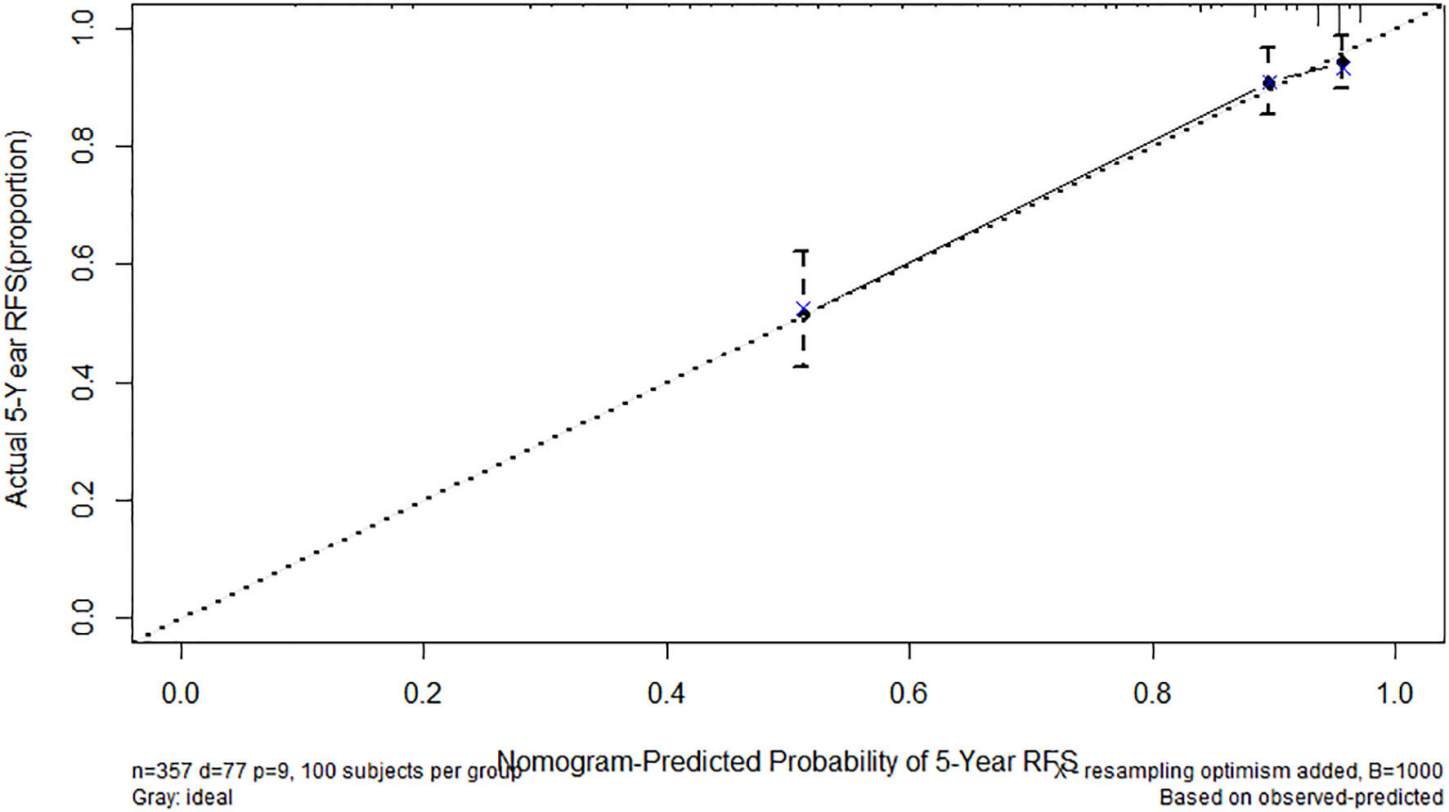
Calibration of nomogram-predicted recurrence-free survival (RFS). Actual 5-years RFS is shown compared with nomogram-Predicted Probability of 5-years RFS.

We established a nomogram that incorporated the significant predictive factors from the multivariate analysis (Figure 4). Tumor size, tumor location, mitotic index, and FARI, which were shown to be independent predictors in the multivariate COX regression analysis, were included in the nomogram. By summing the points of each variable, we can predict the 2-years and 5-years RFS probabilities of each patient. To test its performance, the nomogram was subjected to 1,000 bootstrap resamples for internal validation with a calibration plot. The nomogram-predicted RFS was well-calibrated with the Kaplan-Meier-observed RFS (Figure 5). We further evaluated the effectiveness of the nomogram at predicting the risk of recurrence after resection of primary GISTs. The concordance probability of the nomogram was 0.802 (SE 0·025). Therefore, 80.2% of the time, the nomogram correctly predicted the ordering of the outcome between two randomly selected patients. Subsequently, the predictive ability of the nomogram was compared with the National Institute of Health (NIH) risk classification system. Concordance probabilities of the nomogram were better than NIH risk classification system [0.802 [0.025] vs. 0.737 [0.024], *p* < 0.01].

## Discussion

The current research established the clinical and prognostic value of FARI in 357 patients with GIST who underwent radical resection and developed a novel nomogram predicting recurrence-free survival (RFS). The results showed that the preoperative elevated FARI was a useful indicator of more aggressive tumor phenotype, higher recurrence risk, and poor RFS. Although NLR, PLR, PNI were additionally significant prognostic factors in this study, we established that FARI is the most accurate prognostic factor compared to these serum biomarkers. We developed a nomogram based on tumor size, location, mitotic index, and FARI. The nomogram had a concordance probability of 0.802 (SE 0.025). Concordance probabilities of the nomogram were better than the NIH risk classification system. Few studies have explored the prognostic influence of preoperative FARI in GIST patients undergoing surgery. In the field of GIST surgery, the current research established the clinical value of the preoperative FARI. Hence, FARI could be used as an auxiliary tool to reflect the status of the patient and provide more prognostic information to supplement the postoperative NIH risk classification. The nomogram integrating the NIH risk category and FARI is potentially a more effective tool for predicting RFS.

As a novel Fib/Alb ratio index (FARI) constructed marker, few studies have explored its value in the prognosis of patients with malignant tumors. In a recent study, 1,135 radical esophagectomy patients were retrospectively analyzed, and the results indicated that increased FARI was strictly correlated to poor overall survival in esophageal carcinoma patients (23). Moreover, multivariate analysis indicated that preoperative FARI is an independent predictor of poor prognosis in esophageal carcinoma patients (23). In another study, involving 154 gallbladder cancer (GBC) patients, the findings revealed that patients with a FARI of more than 0.08 have a worse OS compared with patients with a FARI ≤ 0.08 (26). Besides, the multivariate evaluation showed that FARI is an independent predictor of overall survival (26). Herein, our results consistent with the findings of these previous researches indicated that the RFS of the GIST patients with FARI-low is markedly more prolonged compared with patients with FARI-high, implying that the immuno-nutritional status is reduced in the high-risk GIST patients with higher FARI. Furthermore, our multivariate analysis using a stepwise forward method for variable selection showed that the NIH risk category and FARI are independent prognostic indicators for recurrence-free survival.

The 5-years relapse-free survival rate of GIST patients can reach 70.5–79% without imatinib therapy after the operation (27, 28). According to the modified NIH risk classification, about 40% of GIST patients were at high risk and had worse long-term clinical outcomes (28). However, the assessment of GIST recurrence by NIH risk classification is still not accurate enough. Finding more prognostic factors is crucial to guide treatment decision-making. In the current study, we found in the subgroup analysis that in the high-risk subgroup, patients in the FARI-low group had a more prolonged RFS, implying that FARI provides more prognostic information for postoperative NIH risk categories.

Moreover, our results suggest that FARI subdivides high-risk GIST patients in order to speculate GIST recurrence more accurately. Inconsistent with our study, a recent study on soft tissue sarcoma patients, in individual subgroup analysis, indicated that patients in the FARI-low group in the G1/G2 subgroup have a longer OS, but not in the G3 subgroup (29). This could be attributed to the unrecorded adjuvant treatments in the study affecting primary tumor progression. Hence, more studies are required to validate our findings further.

In the present study, we established that an elevated FARI is correlated with larger tumor size, a higher Mitotic index, and advanced NIH risk category but not with tumor location, indicating a more aggressive tumor phenotype. This is the first study, to best of our knowledge exploring the relationship between the preoperative elevated FARI and a more malignant GIST. The results of previously published studies on the relationship between FARI and soft tissue sarcoma (29), hepatocellular cancer (30), and breast cancer (31), also support our conclusions. Furthermore, we established that preoperative elevated FARI is associated with other inflammatory markers, including high PLR and NLR and low PNI. Consistent with our findings, a study of soft tissue sarcoma confirmed that elevated FARI is remarkably associated with inflammatory markers such as NLR and PLR (29). Interestingly, in our study, we discovered that patients with high FARI more likely require open surgery, while patients with low FARI more likely require laparoscopic surgery. This is because surgical procedures recommended in the guidelines (7) are related to GIST size and NIH risk classification, while FARI is highly correlated with GIST size and NIH risk classification.

Previous studies used various cutoff values for FARI. Tan et al. (23) used the ROC curve analysis for the 5-years OS of patients with esophageal cancer and calculated that the best cut-off point of FARI was 0.08. Xu et al. (26) also demonstrated that the best cut-off point of FARI for gallbladder cancer patients is 0.08. Herein, with the help of the ROC curve analysis for the 5-years RFS of patients with GIST, we found that the optimal cutoff point for FARI is 0.08, consistent with the results of the previous studies. However, the optimal cut-off value of FARI in breast cancer is 0.071, which is slightly lower (31). These inconsistencies imply that the optimal FARI cut-off value differs in separate malignancies, although the exact cause and underlying mechanisms remain unclear. However, the discrepancies could be attributed to the distinct biological behaviors of various tumors and gender-associated hormone differences. Hence, more studies should be conducted to validate these findings further.

NLR, PLR, and PNI constitute the frequently used indicators for serum systemic inflammatory response (SIR) and nutritional status. The results of several studies show that NLR, PLR, and PNI are independent prognostic factors in GIST patients (32–35). In the present study, consistent with the findings of these studies, our univariate analysis results revealed that PNI, NLR, and PLR are significant predictors of RFS. Furthermore, FARI has a weak correlation with NLR, PLR, and a weak negative correlation with PNI. Based on the ROC curve, our findings indicated that the AUC value under the ROC curve of FARI is superior to those of the NLR, PLR, and PNI. Compared with other inflammation-based prognostic indices, FARI has a comparable prognostic ability and is more accurate than NLR, PLR, and PNI consistent with the results of a previous study on soft tissue sarcoma (29).

Increasing research evidence shows that hypercoagulable state, malnutrition, and systemic inflammatory response affect tumorigenesis, tumor progression, and metastasis (9, 36). The prognostic value of preoperative FARI is closely linked to patients with malignant tumors (23, 29–31). However, the mechanisms underlying this association remain unknown. Our results are supported by the experimental and clinical studies listed below.

The findings of a recent study involving 91 patients with GIST revealed that elevated fibrinogen levels were related to an increased risk of death or recurrence (22). The increase of fibrinogen could predict the poor long-term prognosis of patients with GIST after the operation. Fibrinogen was a glycoprotein synthesized by hepatocytes in response to pro-inflammatory cytokines. Moreover, fibrinogen is synthesized by malignant tumor cells and participates in the formation of the tumor-reactive extracellular matrix (37). Fibrinogen in the extracellular matrix supports tumor cell migration (37). Besides, fibrinogen promotes tumor progression and metastasis via modulation of tumor cell growth and angiogenesis by binding to many kinds of growth factors (38).

Moreover, fibrinogen binds to fibrinogen receptors, which are expressed in malignant tumor cells and platelets to promote cell-to-cell adhesion. Tumor cells form platelet aggregation in the bloodstream by activating plasma coagulation cascades and direct contact, which increases with the progression and metastasis of the tumor (39). Furthermore, fibrinogen stimulates macrophages to produce high levels of TNF-α (40). Additionally, via the p-AKT/p-mTOR pathway, fibrinogen promotes tumor progression via epithelial-mesenchymal transition (41).

Albumin levels indicate the malnutrition status of an individual as well as implicate the existence of inflammation. The findings of many studies reveal that lower serum albumin leads to a high risk of deterioration and poor prognosis in cancer patients (42). Malnutrition weakens the immune system, increases the chances of infections, and further accelerates the progression of tumors (43). Furthermore, albumin is an essential factor in the systemic inflammatory response. Therefore, albumin levels could be used to reflect tumor prognosis (44).

To sum up, high-risk GIST tumors have a large diameter, a high degree of cell malignancy, high risk of tumor necrosis, and bleeding, resulting in a hypercoagulable state and increased fibrinogen. Increased fibrinogen induces tumor progression and metastasis through various cytokines and signals transduction networks. At the same time, high-risk GIST leads to severe malnutrition and even cachexia, which is characterized by a decrease in serum albumin levels. In contrast, hypoalbuminemia and malnutrition weaken the immune system and further accelerate the progression of tumors. This explains why we observed different prognostic effects of FARI in the high-risk subgroup and the low-risk subgroup in the subgroup analysis. We proposed a nomogram that is clinically simple to use, integrating FARI, and standard NIH risk classification to provide a more accurate prognostic assessment. These results matched those observed in recent studies (6, 45). Further research is needed to improve this nomogram by analyzing more comprehensive prognostic data, and the effectiveness of this model should be evaluated in future clinical applications.

The following limitations should be pointed out in this study. First, this was a single-center retrospective study with a medium sample size. Secondly, due to the relatively long period of sample inclusion and data collection in this study, there could be selection bias concerning diagnosis and clinical treatment. Thirdly, based on the current guidelines, patients who undergo complete resection with a moderate or high risk of recurrence are recommended to receive adjuvant imatinib treatment. However, in this study, patients with moderate or high-risk gastrointestinal stromal tumors treated with imatinib after radical resection were excluded because adjuvant imatinib therapy could significantly prolong RFS.

Nevertheless, this affected the applicability of the conclusions of this study. Fourthly, the GIST included in this study occurred in the stomach, small intestine, colorectal and extra-gastrointestinal tract, and the prognosis of GIST was significantly different in different organs, which may cause statistical bias. Finally, the AUC of FARI was relatively low, so the application value of FARI might be limited. Therefore, in the future, we will design more rigorous prospective studies to verify our preliminary results.

## Conclusions

We established that FARI is an independent predictor of RFS in GIST patients after curative resection, particularly in high-risk patients. FARI is easy to obtain, at low cost and provides accurate prediction, which makes it a potential marker for predicting the prognosis of GIST. This may be helpful for the choice of postoperative treatment for patients with GIST in the future.

## Data Availability Statement

The original contributions presented in the study are included in the article/supplementary material, further inquiries can be directed to the corresponding author.

## Ethics Statement

The studies involving human participants were reviewed and approved by The Beijing Hospital Ethics Committee. The patients/participants provided their written informed consent to participate in this study.

## Author Contributions

GZ and XC set the conceptualization and designed the study. XC wrote the original draft and participated in data curation and data analysis. GZ reviewed and edited the manuscript. JC, TY, and ZL investigated and recorded the clinical data. GZ and JC provided suggestions and useful comments for the revision of the first draft of this article. All authors contributed to the article and approved the submitted version.

## Conflict of Interest

The authors declare that the research was conducted in the absence of any commercial or financial relationships that could be construed as a potential conflict of interest.
